# CARE‐ing for concussions: Development of the Calgary Adapted aRm Ergometer (CARE) exertion test: A physiological alternative to the Calgary Concussion Cycle Test

**DOI:** 10.1113/EP093263

**Published:** 2026-03-19

**Authors:** Jonathan D. Smirl, Joshua J. Burkart, Matthew G. Neill, Jean‐Michel Galarneau, Elizabeth K. S. Fletcher, Joel S. Burma, Nathan E. Johnson, John J. Leddy, Mohammad N. Haider, William M. Adams, Cheri Blauwet, Chantel T. Debert, Carolyn A. Emery

**Affiliations:** ^1^ Cerebrovascular Concussion Laboratory, Faculty of Kinesiology University of Calgary Calgary Alberta Canada; ^2^ Sport Injury Prevention Research Centre, Faculty of Kinesiology University of Calgary Calgary Alberta Canada; ^3^ Human Performance Laboratory, Faculty of Kinesiology University of Calgary Calgary Alberta Canada; ^4^ Hotchkiss Brain Institute University of Calgary Calgary Alberta Canada; ^5^ Integrated Concussion Research Program University of Calgary Calgary Alberta Canada; ^6^ Alberta Children's Hospital Research Institute University of Calgary Calgary Alberta Canada; ^7^ Libin Cardiovascular Institute of Alberta University of Calgary Calgary Alberta Canada; ^8^ UBMD Department of Orthopaedics and Sports Medicine, Jacobs School of Medicine and Biomedical Sciences SUNY at Buffalo Buffalo New York USA; ^9^ Adams Sports Medicine Consulting LLC Colorado Springs Colorado USA; ^10^ Department of Kinesiology University of North Carolina at Greensboro Greensboro North Carolina USA; ^11^ School of Sport, Exercise and Health Sciences, National Centre for Sport and Exercise Medicine Loughborough University Loughborough UK; ^12^ Department of Kinesiology Michigan State University East Lansing Michigan USA; ^13^ Department of Physical Medicine and Rehabilitation Spaulding Rehabilitation/Harvard Medical School Boston Massachusetts USA

**Keywords:** arm crank ergometry, exercise is medicine, exercise physiology, exertion testing, sport‐related concussion, transcranial doppler ultrasound

## Abstract

Aerobic exercise testing helps facilitate recovery post‐concussion. Current protocols (Calgary Concussion Cycling Test: CCCT) are inaccessible for athletes with lower‐body impairments (i.e. Para athletes). This study compared physiological parameters for a novel arm crank test, the Calgary Adapted aRm Ergometer (CARE) test, with the CCCT. Twenty non‐disabled adults (10F:10M, aged 18–51) completed CCCT and CARE to volitional fatigue. Middle cerebral artery velocity (MCAv), heart rate (HR), mean arterial pressure (MAP) and end‐tidal carbon dioxide ($P_{\mathrm{ETC}O_{2}}$) were continuously measured throughout exertion. Bland–Altman with 95% limits‐of‐agreement (LoA), mean differences with effect size measurement and intraclass correlation (ICC) compared CCCT to CARE at 25%, 50%, 75% and 100% of test exertion levels. Test duration was comparable between protocols (*P* = 0.86). HR mean differences between CCCT and CARE ranged from 2 bpm (*P* = 0.45) at 25% to 8 bpm (*P *< 0.05) at 100% effort level (negligible‐to‐medium effect sizes). MCAv mean differences increased from 1.5 cm/s at 25% (*P* = 0.45) to 5.4 cm/s at 100% effort (*P *< 0.05) (small effect sizes). MAP remained stable: 4.1 mmHg at 25% (*P* = 0.11) and 4.8 mmHg at 100% effort (*P* = 0.076) (small effect sizes). $P_{\mathrm{ETC}O_{2}}$ mean differences increased until 75% effort level (2.4 to 4.9 Torr, *P *< 0.05, medium‐to‐large effect size). ICC was good‐to‐moderate across all measurements with 95% LoA widening with increased test intensity. The CARE test represents a promising alternative to traditional lower body exercise tests, demonstrating robust physiological responses and moderate comparability with the CCCT. CARE vs. CCCT differences likely resulted from different total muscle mass engagement between the upper and lower body.

## Abstract (highlights)

- **What is the central question of this study?**
  How does the novel, upper‐body specific, Calgary Adapted aRm Ergometer (CARE) concussion exertion test elicit physiological responses compared to the validated Calgary Concussion Cycle Test (CCCT) in non‐disabled adults for the purpose of enhancing accessibility of post‐concussion exertion testing?
- **What is the main finding and its importance?**
  The CARE test produced robust and moderately physiologically comparable responses across heart rate, cerebral blood velocity and respiratory parameters, particularly at exercise intensities used during clinical concussion assessment. These findings support its use as the first upper‐body specific, post‐concussion exertion protocol, enhancing the inclusivity and equity of concussion care for individuals unable to complete traditional lower‐body tests (i.e. Para athletes).

## 1 INTRODUCTION

Sport‐related concussions (SRC) are a form of mild traumatic brain injury resulting from a direct or indirect impact to the head, neck or body, resulting in forces being transmitted to the brain while engaged in sport or physical activity (Patricios et al., 2023). Estimates suggest approximately 3–4 million SRCs occur annually in North America; however, this number is likely larger due to underreporting (Harmon et al., 2013). SRC generally presents as a heterogeneous combination of cognitive, vestibular and behavioural signs and clinical symptoms, and it is not uncommon for people to experience exercise intolerance following this injury (Haider et al., 2022). While clinical symptom recovery in adults following SRC typically occurs in 10–14 days, about one‐third experience protracted recoveries with symptoms lasting longer than 30 days (Makdissi et al., 2017). Despite the historical treatment of SRC involving complete physical and cognitive rest until symptom resolution (McCrory et al., 2013), it is now well‐established that an early return to sub‐symptom‐threshold aerobic exercise following 24–48 h of relative rest (including light physical activity) reduces recovery by roughly 5 or more days (Leddy et al., 2023) and can reduce the likelihood of persisting post‐concussive symptoms (PPCS) beyond 30 days (Mn et al., 2021). Moreover, those who are exercise intolerant following concussion are at greater risk for protracted recovery, especially in adolescent females (Neill et al., 2025; R. Orr et al., 2021). Thus, it is critically important to evaluate exercise tolerance and prescribe symptom‐informed exercise recommendations following SRC to improve recovery and outcomes (Leddy et al., 2017, 2023; Patricios et al., 2023).

Clinicians often employ incremental exertion testing to help assess symptom profile categorizations of SRCs and to prescribe safe individualized exercise intensity programmes to promote recovery (Leddy et al., 2018, 2023). These exertion tests are designed to gradually increase heart rate (HR), blood pressure, and cerebral blood flow while monitoring concussion symptom exacerbation on an 11‐point Likert scale (Leddy et al., 2018, 2023). The HR threshold (HRt) is achieved when overall concussion symptoms increase by 3 or more points during exertion testing (Leddy et al., 2023). This threshold informs the aerobic exercise training recommendation for recovery following SRC, where exercise is expected to be tolerated without a more‐than‐mild increase in symptoms (Leddy et al., 2023). The Buffalo Concussion Treadmill Test (BCTT) was the first test developed to establish HRt following SRC (Leddy & Willer, 2013). Since then, a physiologically comparable cycle ergometer test, the Calgary Concussion Cycling Test (CCCT), was developed and validated against the BCTT (Miutz et al., 2024).

The BCTT and CCCT are effective exertional tests for ambulatory individuals; however, no validated protocol currently exists for individuals with lower‐body physical impairments. Furthermore, Para sport athletes are significantly under‐represented in the SRC literature despite evidence of similar if not greater risk of SRC (Patricios et al., 2023; Singh et al., 2024; Sobry et al., 2022; Weiler et al., 2021). Para ice hockey, a sport typically played by individuals with lower‐limb impairment, has a high burden of injury, with an SRC rate of 4.5 concussions/1000 athlete exposures (AEs) (Sobry et al., 2022). This is notably higher than rates seen in youth standing hockey (1.2 concussions/1000 AEs) and varsity standing hockey (1.18 concussions/1000 AEs in women and 0.95 concussions/1000 AEs in men) (Pfister et al., 2016; Schick & Meeuwisse, 2003; Sobry et al., 2022). Due to mobility limitations, many athletes in adapted and Para sports are unable to complete existing exertional tests such as the BCTT or CCCT following SRC (Patricios et al., 2023; Weiler et al., 2021). As a result, there remains no validated exertion test to guide sub‐symptom‐threshold exercise prescription following SRC for athletes with lower‐body impairments.

Arm crank ergometry (ACE) is a promising test for Para athletes since it involves only the upper body. ACE is one of the most common forms of aerobic exercise engaged in by persons with lower‐limb impairments offering a safe and effective stimulus to improve cardiovascular fitness (Chiou et al., 2022). Regarding the physiological responses to ACE compared to cycle ergometry exertion exercise, peak volume of oxygen consumption (${\dot{V}}_{O_{2}\mathrm{peak}}$), peak HR (HR_peak_) and peak minute ventilation (${\dot{V}}_{\mathrm{Epeak}}$) are typically lower, while peak rating of perceived exertion (RPE) is comparable across exercise intensities (Hill et al., 2019; J. L. Orr et al., 2013; Schneider et al., 2002). Furthermore, at the same absolute power output, ACE elicits increased ${\dot{V}}_{O_{2}}$, HR, ${\dot{V}}_{E}$ and RPE compared to cycle ergometry, resulting from the large differences in active muscle mass engaged in during upper body (ACE) versus lower body (cycling) exercise (Hill et al., 2019; J. L. Orr et al., 2013; Schneider et al., 2002). However, before there is widespread use of ACE as a means to prescribe aerobic exercise treatment for concussion, a unique exertion test must be developed and validated against current concussion exertion testing protocols (Patricios et al., 2023). Thus, the purpose of this study was to quantify the physiological and metabolic responses during a novel ramping exercise test – the Calgary Adapted aRm Ergometer (CARE) test – versus the CCCT in healthy non‐disabled individuals. We wanted to explore whether CARE elicits increases in physiological and metabolic parameters and can be completed in a similar time as the CCCT.

## 2 METHODS

### 2.1 Ethical approval

The current study was approved by the University of Calgary's Conjoint Health and Research Ethics Board (REB21‐1517), and all procedures and experimental protocols adhered to the guidelines outlined in the *Declaration of Helsinki* (except for study registration in a database) (World Medical Association, 2013). Prior to data collection, written informed consent was obtained and all participants completed a Physical Activity Readiness Questionnaire (PARQ+).

### 2.2 Participants and study design

A convenience sample of 20 healthy adults aged 18–51 (10 female, 10 male) were recruited. Participants were asked about both their assigned biological sex and their self‐identified gender. All participants identified as *cis*‐gendered. Consequently, all data in the investigation were analysed and discussed as they relate to biological sex. Participants were excluded from testing if they had a cardiorespiratory and/or cerebrovascular condition that impaired their ability to attain maximal intensity exercise and/or had sustained a concussion within the past 6 months (Lapointe et al., 2021). None of the recruited participants were excluded based upon these parameters. Participants were instructed to abstain from caffeine, alcohol, vaping, smoking and exercise for a minimum of 8 h before testing to reduce the confounding influence of these substances on the cerebrovasculature and cardiovascular system (Ainslie et al., 2007; Burma et al., 2021; Burma, Copeland et al., 2020, 2020). Furthermore, a previous study found good‐to‐excellent reliability of cerebral blood velocity (CBv) metrics across the menstrual cycle during exercise testing, enabling female participants to participate in this investigation at any stage of the menstrual cycle (Johnson et al., 2023).

### 2.3 Protocols

All exertion testing was conducted in the Cerebrovascular Concussion Laboratory at the University of Calgary at an elevation of 1111 m above sea level. Participants visited the laboratory for two maximal exercise tests (CARE test and CCCT) separated by a minimum 24 h between sessions (average: 10 ± 12 days). The order of the tests was randomly assigned via a random generator. Age and sex were collected via self‐report (Table 1). Height and weight were measured prior to testing, and body mass index (BMI) was calculated (Table 1).

**TABLE 1:** Individual and group level characteristics for participants who completed both the CARE test and CCCT (*n* = 20, 10F:10M), and also each individual's wattage increase and number of stages completed for both the CARE test and CCCT.

	Participant characteristics					CARE		CCCT	
Participant	Sex	Age (years)	Height (cm)	Weight (kg)	BMI (kg/m^2^)	Wattage increase	Stages completed	Wattage increase	Stages completed
1	F	22	161.0	84.0	32.4	5	16	9	19
2	F	23	160.3	68.6	26.7	3	23	8	22
3	F	22	159.5	59.8	23.5	4	24	7	32
4	F	23	156.6	68.7	28.0	2	27	8	18
5	F	24	165.5	70.7	25.8	3	27	8	21
6	F	21	168.2	66.6	23.5	3	25	7	31
7	F	21	160.1	57.4	22.4	3	19	6	24
8	F	51	168.5	59.7	21.0	3	25	7	24
9	F	21	164.0	69.2	25.7	3	18	8	16
10	F	22	166.0	63.5	23.0	5	14	7	26
Female mean (SD)	—	25.0 (9.2)	163.0 (4.0)	66.8 (7.6)	25.2 (3.3)	3 (1.0)	22 (5)	8 (1.0)	23 (5)
11	M	22	176.4	77.0	24.7	6	26	11	26
12	M	30	182.5	81.9	24.6	4	31	11	30
13	M	18	179.3	75.3	23.4	5	24	11	24
14	M	21	185.3	74.6	21.7	5	25	10	26
15	M	29	178.5	101.0	31.7	8	20	14	17
16	M	23	192.0	80.5	21.8	5	31	11	27
17	M	20	177.5	84.5	26.8	4	25	12	21
18	M	51	177.5	71.9	22.8	4	30	10	25
19	M	28	186.3	87.5	25.2	7	21	13	24
20	M	26	170.0	77.5	26.8	7	23	11	25
Male mean (SD)	—	26.8 (9.4)	180.5 (6.2)	81.2 (8.4)	25.0 (3.0)	6 (1)	26 (4)	11 (1.)	25 (4)
Total mean (SD)	—	25.9 (9.1)	171.2 (10.3)	74.0 (10.7)	25.1 (3.1)	5 (2)	24 (5)	10 (2)	24 (4)

*Note*. Female, Male, and Total rows data presented as means (SD). Abbreviations: CARE test, Calgary Adapted aRm Ergometer test; CCCT, Calgary Concussion Cycle Test.

### 2.4 CCCT

The CCCT is a graded exertional test that was completed on a Lode Cycle Ergometer (Corival cpet model, Groningen, Netherlands) (Miutz et al., 2024). This test was developed and validated as a physiological comparison to the industry‐standard BCTT (Leddy & Willer, 2013), allowing for a more inclusive range of modalities to be used for SRC aerobic exercise testing. According to the CCCT protocol, participants begin the test at a wattage relative to their body mass to account for sex differences in relative muscle mass (Miutz et al., 2024). Therefore, females began the test at 0.11 W/kg of body mass and males at 0.14 W/kg with each stage of the CCCT increasing by these individualized wattages (Miutz et al., 2024). Each stage of the CCCT was 1 min in duration until participants reached volitional fatigue. Participants were instructed to maintain a cadence between 70 and 90 revolutions per minute (rpm) (Miutz et al., 2024). Participants were instructed to adjust the seat height to a comfortable position allowing for full‐leg extension during cycling.

### 2.5 Calgary adapted aRm ergometer (CARE) test

The novel CARE test is a graded exertion test performed on a Lode ACE (Angio CPET model, Groningen, Netherlands). The protocol for the CARE test was pilot tested and developed in a separate convenience sample of 16 healthy subjects between the ages of 20 and 29 years (6 females, 10 males; unpublished data). To establish the protocol, participants completed the CCCT and a maximal intensity ACE test separated by at least 24 h and within the same week. Data from pilot testing were used to develop the CARE protocol which based starting wattage and subsequent wattage increases each minute as a function of 10 repetition‐maximum (RM) of bicep curl exercise (Supplementary Material). The 10‐RM bicep curls were selected as a surrogate for body mass that is used in the CCCT due to the variations for Para athletes resulting from medical conditions (i.e. spina bifida, muscular dystrophy, and single or double leg amputations); hence, 10‐RM of bicep curl exercise served as an indicator of upper‐body muscular endurance while also accounting for variations in body mass, allowing the CARE test protocol to employ a familiar exercise for many individuals. While there are concerns about the haemodynamic consequences of resistance exercise exacerbating concussion symptoms (Neill et al., 2024), recent studies employing resistance exercise in a post‐concussion cohort reported no adverse effects indicating that resistance exercise is likely safe post‐concussion in moderation (Hutchison et al., 2023). Moreover, symptom exacerbation during the 10RM bicep curl may decrease the weight used, thus reducing the intensity ramp, which may better titrate exercise tolerance in those who have an increased sensitivity to exercise, though this has yet to be confirmed following SRC. The CARE test started at a wattage equal to 1 watt per 6.7 lbs (3 kg) increase in 10‐RM bicep curl weight (Appendix Table A1) and increased by this same wattage every minute until volitional fatigue. Participants were positioned so that the crankshaft was horizontally aligned with the level of the shoulder joint. Seat distance was self‐adjusted to allow for a slight elbow bend at full arm extension. Throughout the test, participants were instructed to sit upright with their back against the seat and feet flat on the floor in front of them to ensure consistent posture. Participants were asked to maintain a cadence between 70 and 90 rpm due to the known impact of ACE cadence on ${\dot{V}}_{O_{2}\mathrm{peak}}$ (P. M. Smith et al., 2001).

### 2.6 Instrumentation

Mean arterial pressure (MAP), HR, middle cerebral artery velocity (MCAv), ${\dot{V}}_{O_{2}}$_,_ ${\dot{V}}_{E}$ and partial pressure of end‐tidal carbon dioxide ($P_{\mathrm{ETC}O_{2}}$) were continuously measured for the entirety of each exertion test. At the end of each stage, RPE was recorded using the Borg 6–20 scale (Borg, 1982). A polar HR monitor (Polar H10, Kempele, Finland) captured HR continuously. Finger photoplethysmography with a height correction unit fixed to the upper arm was used to assess beat‐to‐beat MAP from the right middle finger corrected to the level of the heart (Finometer NOVA; Finapres Medical Systems, Amsterdam, The Netherlands). Participants were instructed to avoid squeezing the handles of the cycle ergometer and ACE with the finger the cuff was situated on to limit interference with the photoplethysmography signal. Bilateral middle cerebral arteries (MCA) were insonated using transcranial doppler (TCD) ultrasound (DWL USA, Inc., San Juan Capistrano, CA, USA) to quantify CBv as an index of cerebral blood flow through these arteries (Skow et al., 2022). Two 2‐MHz ultrasound probes were positioned over the transtemporal acoustic windows and locked into place using a custom fitting headframe (DWL USA, Inc.). Left and right MCAv were verified to be within 10% of each other before testing, and confirmed via carotid compression (Willie et al., 2011). MCA depth and velocity were recorded for each participant after their first testing session and used as reference values for the second testing day to maximize between‐day reliability of MCAv assessment (Smirl et al., 2015). Specifically, bilateral MCAv signals were continuously acquired throughout each exercise protocol and manually inspected to assess signal quality. For each participant, the MCA signal selected for analysis was the side demonstrating the most stable waveform morphology, minimal motion‐related artifact, and the highest proportion of physiologically plausible, artifact‐free beats across the recording (Weston et al., 2023). Where possible, the same MCA side selected during the first exercise test was also used for the second test to maximize within‐participant consistency and reduce potential side‐related differences in MCAv responses (Smirl et al., 2015; Weston et al., 2023). If the originally selected side demonstrated inferior signal quality during the second test due to technical limitations or excessive artifact, the contralateral MCA signal was selected. ${\dot{V}}_{O_{2}}$ and $P_{\mathrm{ETC}O_{2}}$ were measured from a mixing chamber and breath‐by‐breath, respectively, using a mouthpiece, pneumotach, nose clip and two separate inline gas analysers (ML206; ADInstruments, Colorado Springs, CO, USA). Two‐point calibration (room air: 78% nitrogen, 21% oxygen and 0.04% carbon dioxide; known gas concentration: 5% carbon dioxide and 16% oxygen) and pneumotach calibration with a 3‐litre syringe were utilized prior to each data collection session. All physiological data (${\dot{V}}_{O_{2}}$, $P_{\mathrm{ETC}O_{2}}$, HR, MAP and MCAv) were collected at 1000 Hz and stored offline for analysis using commercially available software (LabChart Pro Version 8, ADInstruments).

### 2.7 Data processing

A mean of datapoints occurring in the final 20 s of each 60‐s stage was extracted in LabChart Pro for each physiological outcome variable for each stage of both exercise tests (CARE and CCCT) and used in the statistical analysis. Due to excessive movement artifact and low signal quality of the photoplethysmography trace, especially during the higher intensities of the CARE test, MAP data for only 10 participants were included in the final analysis. The complete dataset used in this study has been included in the supporting information section (Supporting Information).

### 2.8 Statistical analysis

All statistical analyses were performed in Stata (Version 18.0; StataCorp, College Station, TX, USA) and confirmed by a biostatistician (J.M.G.). All measured parameters were normalized to percentage of test completion to account for individual variability in the number of stages completed between the CARE test and CCCT. Therefore, mean values for HR, MCAv, MAP, $P_{\mathrm{ETC}O_{2}}$, ${\dot{V}}_{O_{2}}$ and ${\dot{V}}_{E}$ were compared during participants’ resting stage (baseline), 25%, 50%, 75% and 100% of test completion (Figures 1 and 5). Mean differences with 95% confidence intervals (CI), intraclass correlation (ICC), and a pseudo Cohen's *d* effect size were calculated to assess the comparability of physiological responses between the two tests (Figures 1 and 5, Tables 2 and 3). Bland–Altman plots for all variables demonstrating the mean bias with upper and lower 95% limits‐of‐agreement (LoA) for baseline, 25%, 50%, 75% and 100% of the exertion test were also calculated (Figures 2, 3, 4, 6, 7, 8). Normality of differences were confirmed using the Shapiro–Wilk test within stage and association between difference values and mean scores evaluated using linear regression (Bland & Altman, 1986). Due to growing concerns in the physiological literature about using binary *P*‐values to analyse results, inferences were made from a combination of 95% CI, *P*‐values, 95% LoA, ICCs, and Cohen's *d* effect sizes (Amrhein et al., 2019). Excellent, good, moderate and poor ICC values were appraised at >0.90, 0.75–0.90, 0.50–0.75 and <0.50, respectively (Koo & Li, 2016). Negligible, small, moderate and large Cohen's *d* effect sizes were appraised at <0.20, 0.20–0.50, 0.50–0.80 and >0.80, respectively (Lakens, 2013). To produce mean differences, ICCs and the pseudo Cohen's *d*, all by percentile of stage completion, a mixed effects model was employed for all outcomes fit with restricted maximum likelihood and Kenward–Rodger degrees of freedom to account for the small number of clusters (participants) (Rabe‐Hesketh & Skrondal, 2022). The model was fit with an interaction term representing CCCT or CARE by percentile of stage completion (baseline, 25%, 50%, 75% and 100%) with an independent residual structure, also by percentile of stage completion, to produce ICCs. To emulate Cohen's *d*, a pooled model standard deviation (by stage) was derived by which the stage/condition specific means were divided. Finally, moderation by sex was evaluated using the Bayesian information criteria (BIC) for all outcomes by adding sex to the two‐way term effectively producing a three‐way interaction but also by producing a residual structure independent by stage, condition and sex consistent with the architecture of the main model.

**FIGURE 1:**
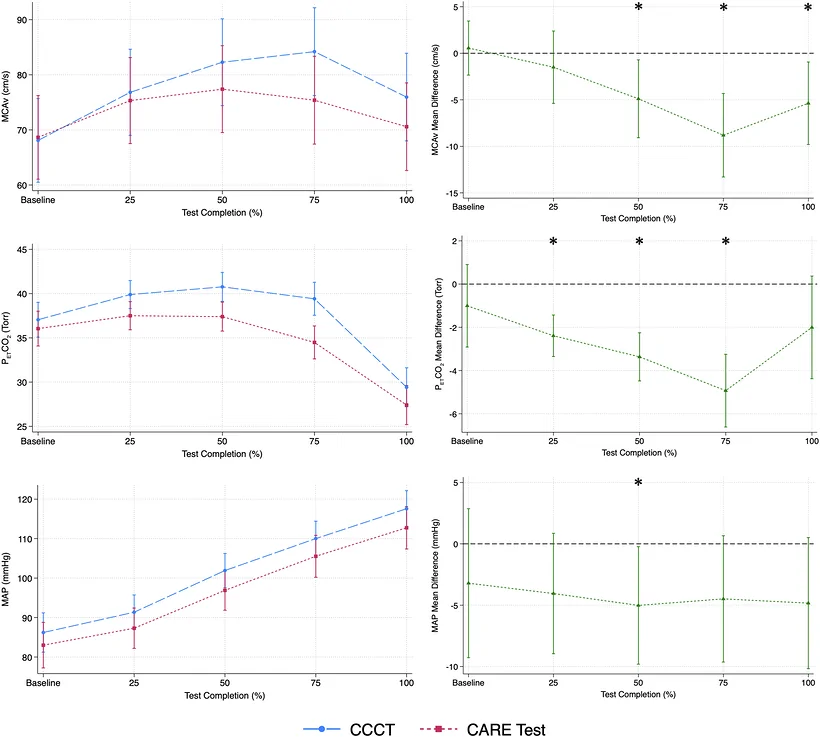
Graphs on left side display the comparison of MCAv, $P_{\mathrm{ETC}O_{2}}$ and MAP responses during the CCCT and the CARE test performed to volitional fatigue for 10 male and 10 female participants who completed both exertional testing sessions. Data are means ± 95% CI for all 20 participants. Graphs on right side display the mean differences between the CCCT and CARE test for MCAv, $P_{\mathrm{ETC}O_{2}}$ and MAP. CARE test, Calgary Adapted aRm Ergometer test; CCCT, Calgary Concussion Cycle Test; MCAv, middle cerebral artery blood velocity; MAP, mean arterial pressure; $P_{\mathrm{ETC}O_{2}}$, partial pressure of end‐tidal carbon dioxide. *Significance (*P *< 0.05).

**TABLE 2:** Mean values and mean differences for MCAv, MAP, $P_{\mathrm{ETC}O_{2}}$ for 20 participants (10F:10 M) who completed both the CARE test and CCCT.

	Condition mean (SD)		Mean difference							
Parameter	CCCT	CARE test	Mean difference	Lower 95%	Upper 95%	*P*	ICC	ICC level	Cohen's *d*	Cohen's *d* level
MCAv (cm/s)										
Baseline	68.1 (16.2)	68.7 (16.9)	−0.6	−3.5	2.4	0.705	0.927	Excellent	0.032	Negligible
25%	76.8 (19.3)	75.3 (17.4)	1.5	−2.4	5.4	0.450	0.876	Good	0.084	Negligible
50%	82.3 (20.0)	77.4 (18.0)	4.9	0.7	9.1	0.022*	0.859	Good	0.272	Small
75%	84.2 (18.5)	75.4 (18.2)	8.8	4.3	13.3	<0.001*	0.841	Good	0.485	Small
100%	76.0 (16.3)	70.6 (18.7)	5.4	0.9	9.8	0.018*	0.845	Good	0.296	Small
MAP (mmHg)										
Baseline	86.2 (10.0)	83.0 (7.6)	3.2	−2.9	9.3	0.301	0.448	Poor	0.303	Small
25%	91.4 (10.5)	87.3 (6.5)	4.1	−0.9	9.0	0.106	0.577	Moderate	0.434	Small
50%	101.9 (10.4)	96.9 (6.9)	5.0	0.2	9.8	0.040*	0.590	Moderate	0.544	Moderate
75%	110.0 (11.2)	105.5 (6.7)	4.5	−0.7	9.6	0.088	0.571	Moderate	0.478	Small
100%	117.6 (10.3)	112.8 (7.2)	4.8	−0.5	10.2	0.076	0.532	Moderate	0.497	Small
$P_{\mathrm{ETC}O_{2}}$ (Torr)										
Baseline	37.1 (4.2)	36.1 (4.0)	1.0	−0.9	2.9	0.302	0.530	Moderate	0.224	Small
25%	39.9 (3.6)	37.5 (3.7)	2.4	1.4	3.4	<0.001*	0.816	Good	0.661	Moderate
50%	40.8 (4.7)	37.4 (3.5)	3.4	2.3	4.5	<0.001*	0.768	Good	0.903	Large
75%	39.4 (5.0)	34.5 (3.3)	4.9	3.3	6.6	<0.001*	0.592	Moderate	1.161	Large
100%	29.4 (5.0)	27.4 (4.2)	2.0	−0.4	4.4	0.098	0.421	Poor	0.398	Small

Data were normalized to percentage of test completion to account for variations in test duration between the CARE test and CCCT. *Significance (*P *< 0.05). CARE test, Calgary Adapted aRm Ergometer test; CCCT, Calgary Concussion Cycle Test; ICC, intraclass correlation; MAP, mean arterial pressure; MCAv, middle cerebral artery blood velocity; $P_{\mathrm{ETC}O_{2}}$, partial pressure of end‐tidal carbon dioxide.

**TABLE 3:** Mean values and mean differences for HR, relative ${\dot{V}}_{O_{2}}$, and ${\dot{V}}_{E}$ for 20 participants (10F:10M) who completed both the CARE test and CCCT.

	Condition mean (SD)		Mean difference							
Parameter	CCCT	CARE test	Mean difference	Lower 95%	Upper 95%	*P*	ICC	ICC Level	Cohen's *d*	Cohen's *d* level
HR (bpm)										
Baseline	82 (13)	80 (11)	2	−2	7	0.317	0.685	Moderate	0.178	Negligible
25%	108 (14)	106 (16)	2	−3	7	0.454	0.627	Moderate	0.145	Negligible
50%	136 (14)	129 (19)	7	1	14	0.023*	0.531	Moderate	0.494	Small
75%	162 (14)	154 (17)	8	2	14	0.009*	0.561	Moderate	0.549	Moderate
100%	184 (12)	176 (13)	8	2	13	0.004*	0.610	Moderate	0.574	Moderate
Relative ${\dot{V}}_{O_{2}}$ (mL/kg/min)										
Baseline	7.0 (3.0)	6.0 (2.3)	1.0	−0.7	2.7	0.236	0.519	Moderate	0.260	Small
25%	15.6 (3.4)	11.9 (2.3)	3.7	2.7	4.7	<0.001*	0.744	Moderate	1.138	Large
50%	22.8 (5.5)	16.4 (3.3)	6.5	4.8	8.2	<0.001*	0.508	Moderate	1.642	Large
75%	31.7 (6.3)	22.3 (4.5)	9.4	7.3	11.5	<0.001*	0.408	Poor	2.128	Large
100%	41.0 (8.6)	29.2 (6.8)	11.8	8.0	15.5	<0.001*	0.175	Poor	1.746	Large
${\dot{V}}_{E}$ (L/min)										
Baseline	20.0 (7.4)	18.4 (4.0)	1.7	−2.1	5.4	0.381	0.441	Poor	0.211	Small
25%	31.8 (12.2)	26.0 (4.4)	5.8	1.0	10.6	0.017*	0.316	Poor	0.622	Moderate
50%	47.4 (16.7)	36.9 (7.5)	10.5	4.4	16.6	0.001*	0.222	Poor	0.940	Large
75%	67.9 (23.2)	52.5 (11.9)	15.4	5.9	24.9	0.001*	0.106	Poor	0.953	Large
100%	104.6 (31.7)	81.5 (23.5)	23.2	7.6	38.7	0.003*	0.042	Poor	0.905	Large

Data were normalized to percentage of test completion to account for variations in test duration between the CARE test and CCCT. *Significance (*P *< 0.05). CARE test, Calgary Adapted aRm Ergometer test; CCCT, Calgary Concussion Cycle Test; ICC, intraclass correlation; HR, heart rate; Relative ${\dot{V}}_{O_{2}}$, relative volume of oxygen consumption; ${\dot{V}}_{E}$, minute ventilation.

**FIGURE 2:**
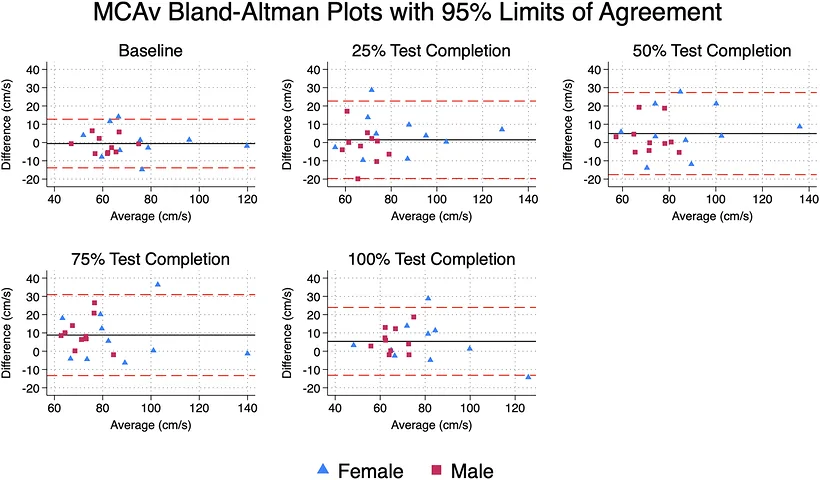
Bland–Altman Plots with 95% limits of agreement for middle cerebral artery velocity (MCAv, cm/s) between the Calgary Concussion Cycle Test and the Calgary Adapted aRm Ergometer (CARE) test. Females are represented by the blue triangles, while males are represented by the red squares.

**FIGURE 3:**
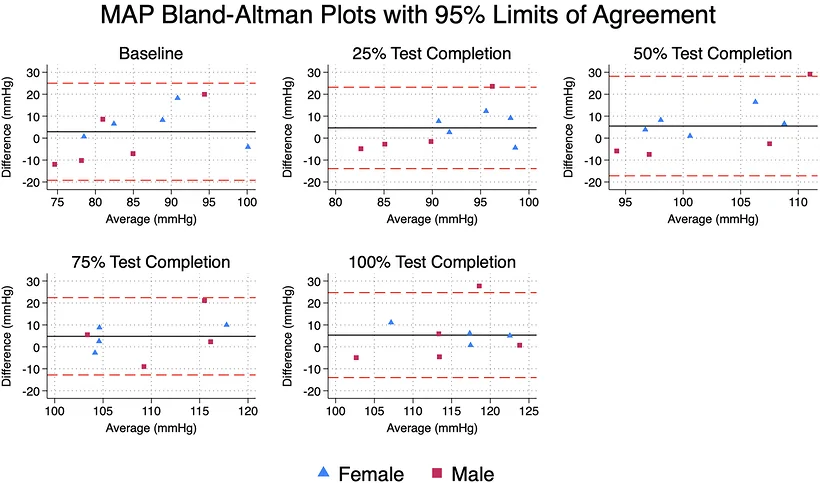
Bland–Altman plots with 95% limits of agreement for mean arterial pressure (MAP, mmHg) between the Calgary Concussion Cycle Test and the Calgary Adapted aRm Ergometer (CARE) test. Females are represented by the blue triangles, while males are represented by the red squares.

**FIGURE 4:**
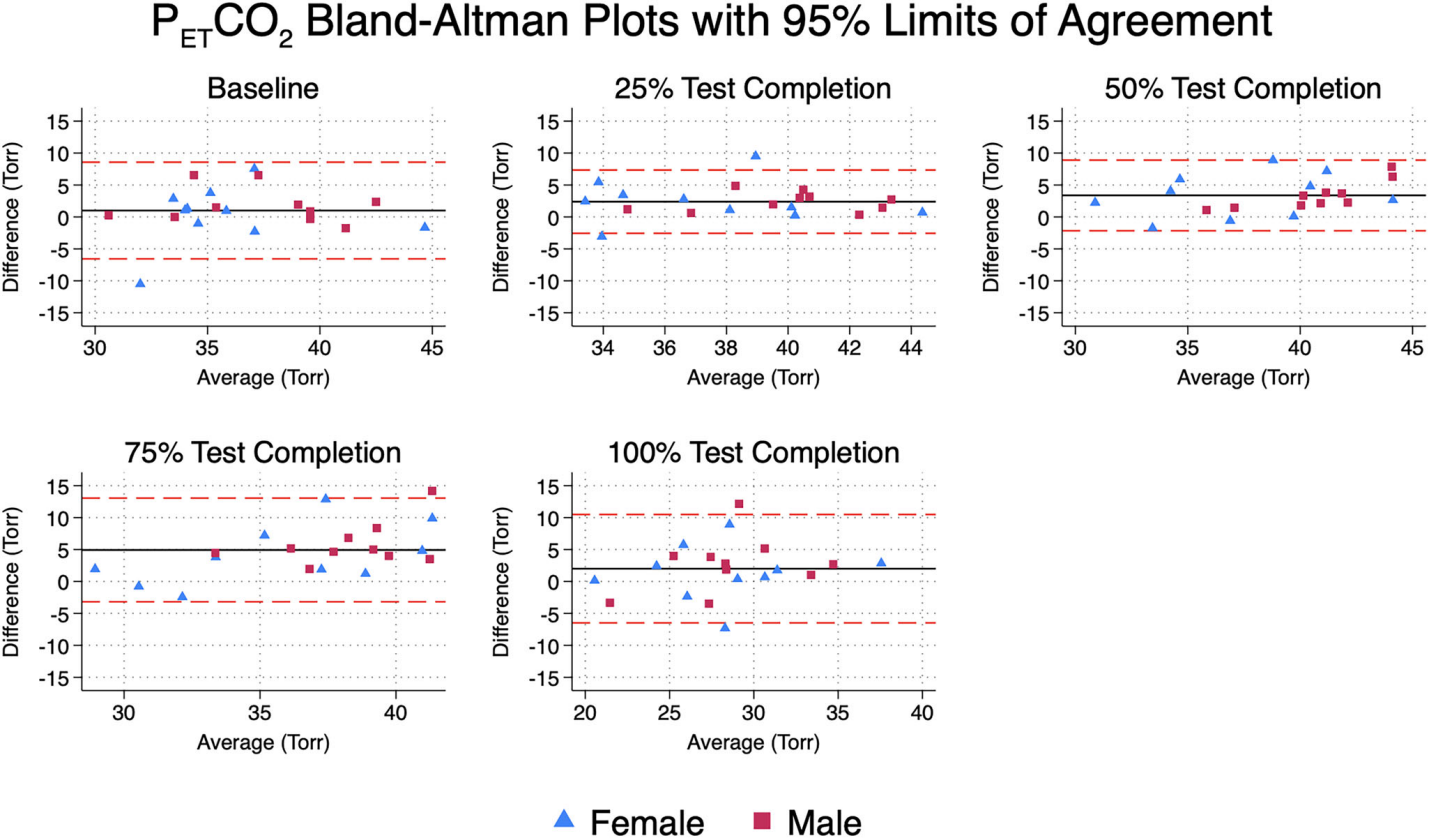
Bland–Altman Plots with 95% limits of agreement for partial pressure of end‐tidal carbon dioxide ($P_{\mathrm{ETC}O_{2}}$, Torr) between the Calgary Concussion Cycle Test and the Calgary Adapted aRm Ergometer (CARE) test. Females are represented by the blue triangles, while males are represented by the red squares.

## 3 RESULTS

Twenty healthy adults (10 female, 10 male; ages 18–51) took part in exercise testing for the current investigation (Table 1). All participants completed both exertion tests and were included in the analysis. Table 1 highlights the demographics of the participants. The average numbers of stages completed was 24 ± 5 (females, 22 ± 5; males, 26 ± 4) on CARE and 24 ± 4 on CCCT (females, 23 ± 5; males, 25 ± 4; Table 1), which was not different between tests (*t* = −0.14, *P* = 0.89). Furthermore, average RPE at 100% of test completion (the final stage) for each exertion test was 19 (CARE) and 20 (CCCT) for females, and 20 on both CARE and CCCT for males. No significant differences were observed when comparing baseline physiological values prior to testing sessions (Tables 2 and 3). Difference distributions were mostly normal, and differences unassociated with mean scores within stage on the various outcome measures. Minute ventilation, however, had differences that were highly associated with means in stages 1–4 (*P* < 0.005) and significant Shapiro–Wilk tests on stages 2–5 (*P* < 0.017). Sex acted as a moderator (lower BIC) for HR and negligibly for ${\dot{V}}_{E}$ but was not retained prioritizing parsimony and consistency across all outcomes.

### 3.1 Physiological parameters (MCAv, MAP and $P_{\mathrm{ETC}O_{2}}$)

The mean difference in MCAv between the CARE and CCCT increased progressively until 75% of test completion, ranging from 1.5 cm/s (95% CI: −2.4, 5.4; *P* = 0.022, ICC = 0.859 [good], Cohen's *d* = 0.272 [small]) at 25% test completion and peaking at 8.8 cm/s (95% CI: 4.3, 13.3; *P* < 0.001, ICC = 0.841 [good], Cohen's *d* = 0.485 [small]) at 75% of test completion (Figure 1, Table 2). At 100% of test completion the mean difference in MCAv between the CCCT and CARE test was 5.4 cm/s (95% CI: 0.9, 9.8; *P* = 0.018, ICC = 0.845 [good], Cohen's *d* = 0.296 [small]) (Table 2). ICC and Cohen's *d* for MCAv were consistently good and small, respectively, for the duration of the tests (Table 2). Bland–Altman analysis showed that the 95% LoA widened from 25% to 75% test completion: −19.7 to 22.7 cm/s and −13.3 to 31.0 cm/s, respectively (Figure 2). At 100% test completion, the LoA was narrower compared to 75%: −13.2 to 23.9 cm/s (Figure 2).

MAP mean differences across the duration of both exercise tests remained stable between the CARE and CCCT, ranging from 4.1 mmHg (95% CI: −0.9, 9.0; *P* = 0.106, ICC = 0.577 [moderate], Cohen's *d* = 0.434 [small]) at 25% test completion to 5.0 mmHg (95% CI: 0.2, 9.8; *P* = 0.040, ICC = 0.590 [moderate], Cohen's *d* = 0.544 [moderate]) at 50% test completion and 4.8 mmHg (95% CI: −0.5, 10.2; *P* = 0.076, ICC = 0.532 [moderate], Cohen's *d* = 0.497 [small]) at 100% test completion (Figure 1, Table 2). ICC was consistently moderate while Cohen's *d* was appraised to be moderate at 50% test completion with all other comparisons appraised as small (Table 2). Bland–Altman analysis for MAP also displayed relatively stable 95% LoA across test completion ranging from −13.9 to 23.2 mmHg, −12.8 to 22.4 mmHg and −14.0 to 24.7 mmHg at 25%, 75% and 100% test completion, respectively (Figure 3).

The mean difference in $P_{\mathrm{ETC}O_{2}}$ increased from 2.4 Torr (95% CI: 1.4, 3.4; *P* < 0.001, ICC = 0.816 [good], Cohen's *d* = 0.661 [moderate]) to 4.9 Torr (95% CI: 3.3, 6.6; *P* < 0.001, ICC = 0.592 [moderate], Cohen's *d* = 1.16 [large]) at 25% and 75% test completion, respectively (Figure 1, Table 2). At 100% test completion the mean difference was 2.0 Torr (95% CI: −0.4, 4.4; *P* = 0.098, ICC = 0.421 [poor], Cohen's *d* = 0.398 [small]). ICC was good at 25% and 50% test completion, moderate at 75% test completion and poor at 100% test completion (Table 2). Cohen's *d* effect size was moderate at 25% test completion, large at 50% and 75% test completion, and small at 100% test completion (Table 2). Bland–Altman analysis displayed a widening of the upper 95% LoA from 25% to 75% test completion: –2.6 to 7.3 Torr and –3.2 to 13.0 Torr respectively (Figure 4). At 100% test completion the 95% LoA was –6.5 to 10.5 Torr (Figure 4).

### 3.2 Metabolic parameters (HR, relative ${\dot{V}}_{O_{2}}$_,_ ${\dot{V}}_{E}$)

The mean difference in HR between the CARE test and CCCT progressively increased with exercise intensity, ranging from 2 bpm (95% CI: −3, 7; *P* = 0.45, ICC = 0.63 [moderate], Cohen's *d* = 0.15 [negligible]) to 8 bpm (95% CI: 3, 13; *P* = 0.004, ICC = 0.61 [moderate], Cohen's *d* = 0.57 [moderate]) at 25% and 100% test completion, respectively (Figure 5 and Table 3). ICC for HR was moderate across all intensity levels, while Cohen's *d* effect sizes ranged from negligible to moderate (Table 3). Bland–Altman analysis for HR displayed widening 95% LoA from 25% (−22 to 26 bpm) to 50% (−25 to 40 bpm) before appearing to narrow at 100% test completion (−7 to 24 bpm) (Figure 6).

**FIGURE 5:**
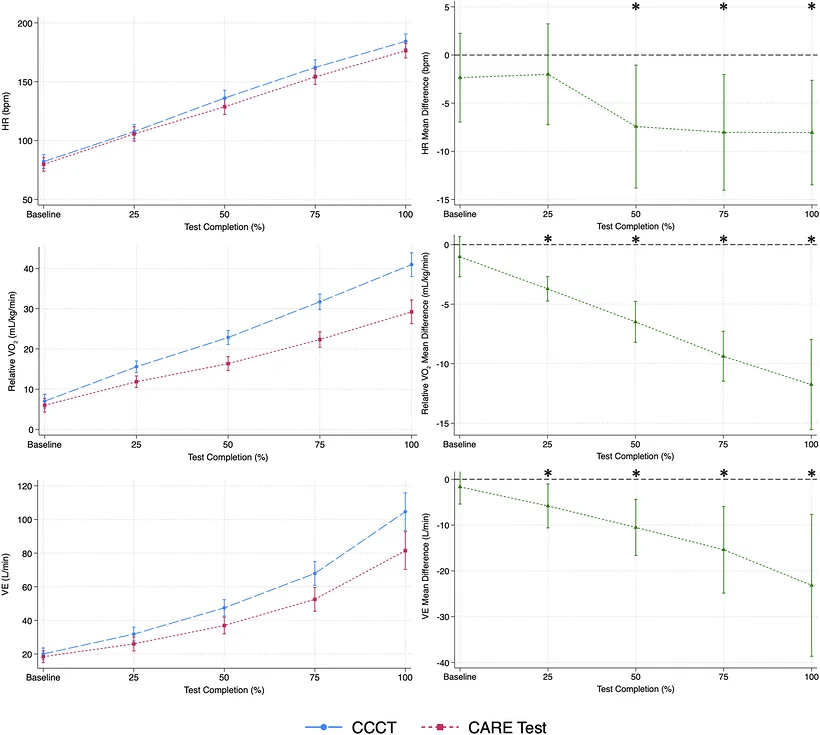
Graphs on left side display the comparison of HR, Relative ${\dot{V}}_{O_{2}}$ and ${\dot{V}}_{E}$ responses during the CCCT and the CARE test performed to volitional fatigue for 10 male and 10 female participants who completed both exertional testing sessions. Data are means ± 95% CI for all 20 participants. Graphs on right side display the mean differences between the CCCT and CARE test for HR, relative ${\dot{V}}_{O_{2}}$ and ${\dot{V}}_{E}$. CARE test, Calgary Adapted aRm Ergometer test; CCCT, Calgary Concussion Cycle Test; HR, heart rate; relative ${\dot{V}}_{O_{2}}$, relative volume of oxygen consumption; ${\dot{V}}_{E}$, minute ventilation. *Significance (*P *< 0.05).

**FIGURE 6:**
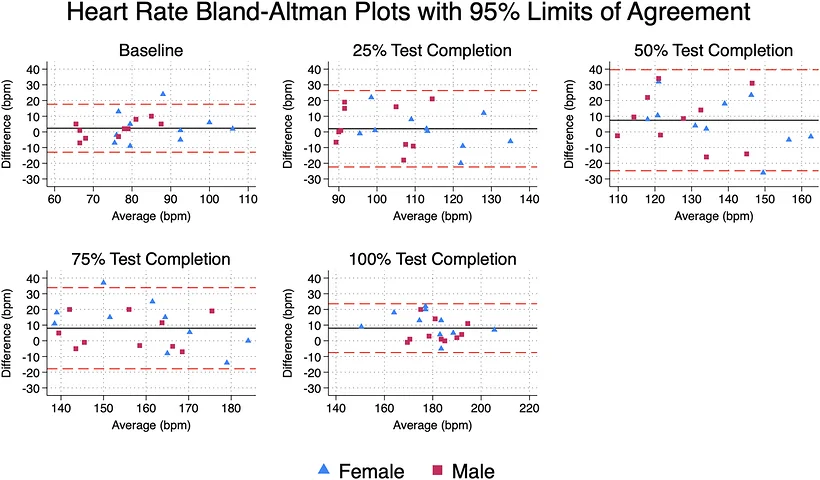
Bland–Altman Plots with 95% limits of agreement heart rate (bpm) between the Calgary Concussion Cycle Test and the Calgary Adapted aRm Ergometer (CARE) test. Females are represented by the blue triangles, while males are represented by the red squares.

When comparing protocols as percentage of test completion progressed, mean differences in relative ${\dot{V}}_{O_{2}}$ increased (Figure 5). At 25% test completion the mean difference was 3.7 mL/kg/min (95% CI: 2.7, 4.7; *P* < 0.001, ICC = 0.744 [moderate], Cohen's *d* = 1.138 [large]) while at 75% test completion it was 9.4 mL/kg/min (95% CI: 7.3, 11.5; *P* < 0.001, ICC = 0.408 [poor], Cohen's *d* = 2.128 [large]) (Table 3). Finally at 100% test completion, the mean difference was 11.8 mL/kg/min (95% CI: 8.0, 15.5; *P* < 0.001, ICC = 0.175 [poor], Cohen's *d* = 1.746 [large]) (Table 3). ICC was moderate for 25% and 50% test completion and poor for 75% and 100% test completion. Cohen's *d* effect sizes were large for every comparison (Table 3). Bland–Altman analysis revealed a progressive increase in both lower and upper 95% LoA, ranging from −1.1 to 8.6 mL/kg/min at 25%, 2.4 to 16.4 mL/kg/min at 75% test completion, and 4.6 to 18.9 mL/kg/min at 100% test completion (Figure 7).

**FIGURE 7:**
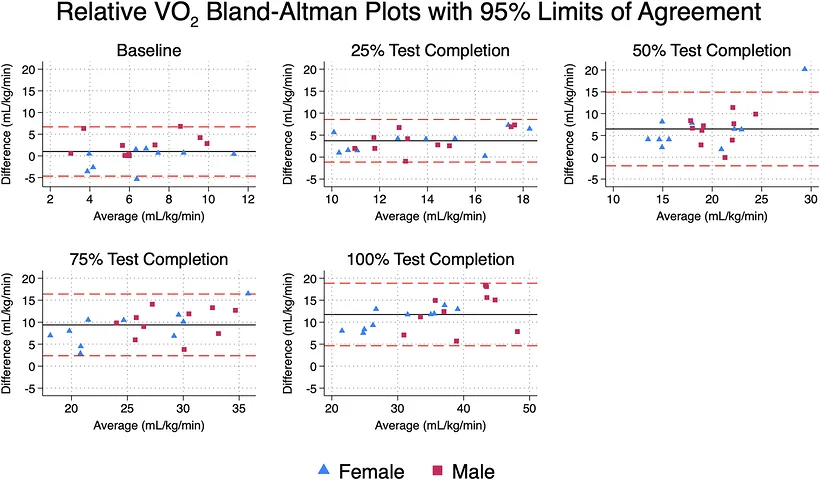
Bland–Altman Plots with 95% limits of agreement for relative volume of oxygen consumption (${\dot{V}}_{O_{2}}$) (mL/kg/min) between the Calgary Concussion Cycle Test and the Calgary Adapted aRm Ergometer (CARE) test. Females are represented by the blue triangles, while males are represented by the red squares.

Like relative ${\dot{V}}_{O_{2}}$, the mean difference in ${\dot{V}}_{E}$ also increased with test duration (Figure 5, Table 3). At 25% test completion the mean difference was 5.8 L/min (95% CI: 1.0, 10.6; *P* = 0.017, ICC = 0.316 [poor], Cohen's *d* = 0.622 [moderate]) while at 100% test completion it was 23.2 L/min (95% CI: 7.6, 38.7; *P* = 0.003, ICC = 0.042 [poor], Cohen's *d* = 0.905 [large]) (Table 3). ICC was considered poor at every comparison while Cohen's *d* effect size was large except for 25% test completion when it was considered moderate (Table 3). Bland–Altman analysis for ${\dot{V}}_{E}$ displayed a progressive widening of the 95% LoA with increasing exercise intensity (Figure 8). At 25% the LoA ranged from −18.4 to 30.0 L/min and expanded to –28.3 to 59.1 L/min at 75% test completion. 100% test completion displayed the largest limits: –25.5 to 71.8 L/min.

**FIGURE 8:**
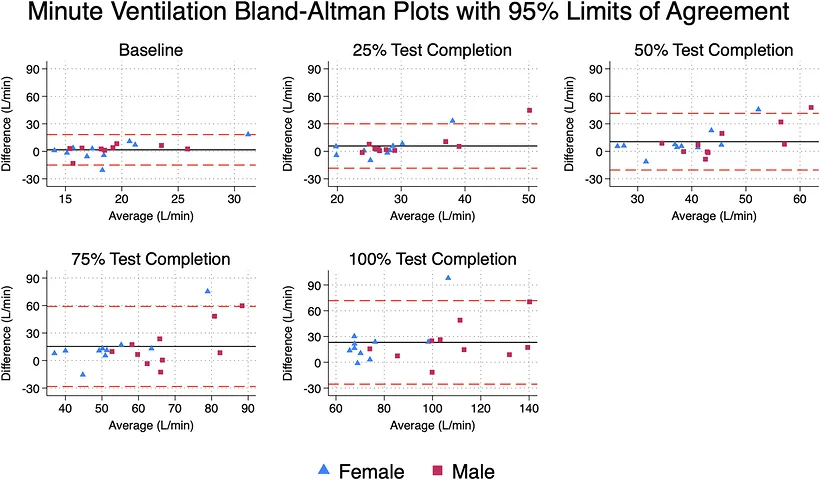
Bland–Altman Plots with 95% limits of agreement for minute ventilation (L/min) between the Calgary Concussion Cycle Test and the Calgary Adapted aRm Ergometer (CARE) test. Females are represented by the blue triangles, while males are represented by the red squares.

## 4 DISCUSSION

The current study compared physiological responses of a novel ACE exertion test (CARE) with a previously validated concussion management cycling exertion test (CCCT). HR, MCAv, MAP, $P_{\mathrm{ETC}O_{2}}$, relative ${\dot{V}}_{O_{2}}$ and ${\dot{V}}_{E}$ assessed at quartiles of total test duration were most comparable between the CARE and CCCT tests during lower exercise intensity (25% and 50% of test completion), with differences becoming more pronounced as participants reached volitional fatigue. (Figures 1 and 2, Tables 2 and 3). Bland–Altman analyses complemented these comparisons by revealing larger individual variability in physiological responses, particularly at increased exercise intensities (Figures 2, 3, 4, 6, 7, 8). Despite the same techniques used for assessment of each parameter, the heterogeneity of physiological responses to the different exertion tests is in line with the broader exercise physiology literature (K. J. Smith & Ainslie, 2017; W.D. McArdle et al., 2010). Previous studies have found lower ${\dot{V}}_{O_{2}\mathrm{peak}}$, lower HR_peak_ and lower ${\dot{V}}_{\mathrm{Epeak}}$ during ACE tests compared to cycling, with the differences becoming more pronounced at increased exercise intensities (Hill et al., 2019; J. L. Orr et al., 2013) (Figure 5, Table 3). This difference in response is mainly attributed to the lower metabolic costs of upper‐body exercise (CARE) versus lower‐body exercise (CCCT), with upper‐body exercise involving much smaller lean muscle mass as compared to the lower limbs (J. L. Orr et al., 2013). Furthermore, past research has also found ACE to cause augmented type II muscle fibre recruitment compared to cycling exercise (J. L. Orr et al., 2013; Schneider et al., 2002). To the authors’ knowledge, there are no previous studies comparing the MCAv response between ACE and cycling (Figure 1, Table 2). We found smaller increases in MCAv during ACE compared to cycling exercise in this sample of non‐disabled adults (Table 2, Figure 2). However, the CARE test elicited the expected parabolic MCAv response, typical for incremental exercise tests performed to volitional fatigue on a cycle ergometer (K. J. Smith & Ainslie, 2017; Tan et al., 2014) (Figure 1). Although smaller increases in MCAv were observed during the CARE test, ACE increased MCAv at mild‐to‐moderate workloads, an important component for assessing concussion exertion testing and for informing clinical management strategies (Burma et al., 2023; Haider et al., 2022; Johnson et al., 2023; Tan et al., 2014) (Figure 1).

The CARE test was developed and adapted based on the CCCT (a physiological comparison to the BCTT; Leddy & Willer, 2013), which accounts for biological sex and body mass (kg) by adjusting the incremental workload with sex‐specific formulas (Miutz et al., 2024). The CARE test builds upon this principle by tailoring individualized wattage increases based on the weight achieved during a 10‐RM bicep curl test (Appendix Table A1). Bicep curl exercise as an index for upper‐body strength and endurance in CARE increases workload tailored to an individual's specific capabilities rather than a less physiologically informed surrogate for muscular aerobic capacity based on body mass. This produced similar testing durations and stages completed compared with the CCCT, indicating that the 10‐RM protocol accounted well for inter‐individual variability in upper‐body muscular endurance. Furthermore, since body mass reported by Para athletes is known to vary greatly depending on individual circumstances (such as the absence of one or both lower limbs), its use as an indicator for workload increases was not feasible since it may not be representative of their strength and endurance capabilities.

### 4.1 Physiological and metabolic comparison of the CARE test and CCCT

While differences in physiological and metabolic values were observed at matched intensity levels when comparing CARE and CCCT (Tables 2 and 3), robust response profiles were elicited for each parameter during the two exercise modalities (Figures 1 and 2). Notably, similar increases in HR were observed from baseline values to 25% test completion and from 25% to 50% of test completion for both the CARE and CCCT (Figure 5, Table 3) with 95% LoA becoming wider, highlighting individual response variability between protocols (Figure 6). As exercise intensity increased past 50% of test completion, HR differences became more pronounced with participants’ HR_peak_ on average 8 bpm lower during the CARE test, aligning with previous literature (Hill et al., 2019; J. L. Orr et al., 2013) (Table 3). This is important since HR measurement is currently a cornerstone of post‐concussion exercise prescriptions, and protocols such as the BCTT rely on this metric to determine the HRt at which concussive symptoms are exacerbated (Leddy et al., 2018, 2023; Mn et al., 2021). The HRt is used to prescribe aerobic exercise, which has been found to facilitate physiological recovery (Leddy et al., 2018, 2023). The HR response observed during the CARE test, particularly its similarity to the CCCT at low to moderate intensities where exercise prescriptions are developed in the early stages of concussion management (Leddy et al., 2018), supports the CARE test's potential utility as an inclusive and accessible modality for determining a Para athlete's HRt (Figure 5). Additionally, MAP was similar between CARE and CCCT with mean differences and 95% LoA remaining relatively small and stable throughout the duration of the tests (Figures 1 and 3, Table 2).

There is solid evidence of autonomic dysfunction after concussion (Pelo et al., 2026). Exercise improves abnormal autonomic nervous system function post‐concussion so it is reassuring that MAP responses were similar during ACE compared to cycling (Figures 1 and 3, Table 2). Furthermore, cerebrovascular regulation is perturbed following SRC (Neill et al., 2024), which has been associated with exercise intolerance and prolonged recovery (Clausen et al., 2016). Thus, it is critical that a clinical exercise test provide a sufficient homeostatic challenge to the cerebrovasculature. While a robust MCAv response profile was observed for both CARE and CCCT, the values at each comparison level were slightly lower during the CARE test, aligning with the decreased $P_{\mathrm{ETC}O_{2}}$ values also observed (K. J. Smith & Ainslie, 2017) (Figure 1, 2 and 4, Table 2). Carbon dioxide is a potent vasodilator and $P_{\mathrm{ETC}O_{2}}$ often is a main contributor for the MCAv response during exercise (K. J. Smith & Ainslie, 2017; Tan et al., 2014). Relevant to concussion, $P_{\mathrm{ETC}O_{2}}$ has been found to increase disproportionally to exercise intensity leading to elevations in MCAv during mild‐to‐moderate exertion, which may be associated with exacerbated symptoms and exercise intolerance not observed in uninjured individuals (Leddy et al., 2018; Siedlecki et al., 2018). While ACE produced a blunted $P_{\mathrm{ETC}O_{2}}$ and MCAv response to exercise compared to cycling, the authors believe that these responses are adequate to induce exercise‐related increases in MCAv for the purpose of assessing exercise intolerance post‐concussion (Leddy et al., 2018) (Figure 1, 2 and 4, Table 2). ACE may also be of benefit to non‐disabled individuals with severe exercise‐related symptom exacerbation or persisting symptoms (Haider et al., 2021), enabling them to engage in aerobic exercise at intensities previously limited by the greater increases in $P_{\mathrm{ETC}O_{2}}$ and MCAv observed during cycling or treadmill exercise (Figures 2 and 4). Consistent with previous findings, the CARE test elicited lower relative ${\dot{V}}_{O_{2}}$ and ${\dot{V}}_{E}$ compared to the CCCT at all comparison points during exertion (Hill et al., 2019; J. L. Orr et al., 2013) (Figure 5, 7 and 8, Table 3). These were expected findings as ACE elicits a smaller increase in oxygen utilization, is less biomechanically efficient, and recruits a lower total active muscle mass compared to lower body exercise (Hill et al., 2019; J. L. Orr et al., 2013; Schneider et al., 2002). Bland–Altman analyses highlighted the individual variability within responses between CARE and CCCT, with individual variability generally becoming more pronounced (widening 95% LoA) as test intensity progressed. This variability was expected given the smaller muscle mass recruited during ACE and participants limited familiarity with upper‐body endurance exercise. The variability in individual responses reinforces the importance of individualized interpretation of post‐concussion exertion test data, consistent with the broader recognition that concussion symptomology, exercise capacity post‐injury, and recovery is highly variable between individuals. Altogether, despite increasing differences in the physiological response to ACE and cycling exercise as exercise intensity increased, the robust responses and relatively similar physiological trajectories observed during mild‐to‐moderate intensity exercise (around 25% and 50% test completion) highlight the potential of CARE as a viable option for post‐concussion exertion testing in populations where lower body exercise is not possible.

### 4.2 Clinical implications and future directions

Currently, no post‐concussion exertion test exists that is sensitive to the impairments and limitations of athletes unable to perform lower‐body exercise. Therefore, there is current need for a physiologically informed upper‐body specific exertion test that approximates traditional concussion exertion tests (BCTT, CCCT). The CARE test addresses this significant gap in concussion research, marking a crucial advancement toward more accessible, inclusive and equitable concussion care, and sports injury research. As participation rates in adapted sports continue to grow, concussion risk and rates will likely rise as well, compounded by the lack of sport injury and concussion prevention research specific to Para athletes (Weiler et al., 2021). As such, the CARE test has promising clinical utility as a tool for identifying exercise intolerance and symptom exacerbation thresholds in individuals where traditional lower‐body tests may not be feasible. Importantly, this accessible protocol may also help guide tailored aerobic exercise prescriptions which are physiologically informed to facilitate recovery in Para athletes, those with concurrent lower‐limb injuries, or individuals experiencing dizziness or symptom provocation limiting their ability to perform treadmill or bike‐based exertion tests (Leddy et al., 2018). Given the observed inter‐individual variability in physiological responses between CARE and CCCT (Figures 2, 3, 4, 6, 7, 8) and the individualized nature of concussion recovery, clinical interpretation should be tailored to each participant's unique physiological and symptom response profile. This becomes even more relevant in the context of Para athletes, where impairment‐specific considerations may further influence physiological response profiles and recovery trajectories though further work is needed to assess this (Patricios et al., 2023; Weiler et al., 2021). Therefore, before clinical application of the CARE test, future research should explore whether similar patterns of physiological responses and variability are present in individuals with greater upper‐body endurance training (e.g., rowers, swimmers) and compare between Para athletes and non‐disabled participants. Such work will enhance the generalizability and clinical utility of the CARE test and help establish its role within broader concussion management strategies. The present study represents a necessary first step toward promoting more inclusive and equitable post‐concussion recovery strategies, providing a strong foundation for future research to expand upon to ensure SRC research remains representative of the diversity observed in the larger sporting community.

### 4.3 Limitations

This study is limited by a relatively small sample size (*n* = 20); however, an equal number of males and females were tested with outcomes compared within participants as each completed both exertion tests. Furthermore, the validation of previous post‐concussion exertion tests such as the CCCT (Miutz et al., 2024) and Buffalo Concussion Bike Test (Haider et al., 2019) used similar numbers of participants (*n* = 17 and *n* = 20, respectively), supporting the appropriateness of the current study's sample size. Moreover, the sample consisted primarily of youth/young adults (<30 years of age) with the inclusion of two participants >50 years. While the inclusion of these participants only modestly improves generalizability, future validation of the CARE test for use in middle aged and older adults using a representative sample in that age is required. Furthermore, future research with more diverse cohorts, especially adolescent athletes, Para sport athletes, participants with greater upper‐body endurance familiarity, and concussed participants is warranted to confirm the present findings and support the clinical utility of the CARE test across a broader population. It is also necessary to compare the physiological responses of Para athletes with various impairments to those of non‐disabled athletes to improve the generalizability of the CARE test across different ages, impairments and fitness levels. These differences are likely to impact the physiological responses to exertion testing in general and may also influence agreement between the CARE and CCCT. Future research should include both concussed and non‐concussed participants from each group to better understand how injury status may influence test responses.

Another limitation of the current study was the use of finger photoplethysmography to measure MAP during the CARE test. While finger photoplethysmography can be collected during exercise when the finger can be held in a relatively stable position (treadmill and bike exercise) (Burkart et al., 2024; Miutz et al., 2024), its use during ACE is limited by the signal interference caused by the continuous upper body cycling motion. In the present study, significant signal artifact made MAP recording during the CARE test for 10 participants not possible. In addition, the use of TCD in the present study as an index of cerebral blood flow is limited by the technology's assumptions (Skow et al., 2022). As TCD cannot quantify vessel diameter, MCAv is measured under the assumption vessel diameter is constant (Ainslie & Hoiland, 2014). While vessel diameter changes minimally within ∼8 Torr of eucapnia (Ainslie & Hoiland, 2014), in the present study, $P_{\mathrm{ETC}O_{2}}$ during the late stages of both tests (near volitional fatigue) decreased beyond 8 Torr of eucapnia which could result in a slight underestimation of cerebral blood flow. Despite this, numerous studies have utilized TCD to measure cerebrovascular responses to exercise due to its temporal superiority and robustness to movement artifacts (Burkart et al., 2024; Miutz et al., 2024; Willie et al., 2011). While participants were asked to abstain from the consumption of caffeine, alcohol and nicotine, habitual use was not explicitly collected. Thus, effects of chronic consumption of these confounding influences, or withdrawal may have influenced results (Murray et al., 2018).

Another limitation of the present study was the determination of workload using the 10‐RM bicep curl. Familiarity with resistance exercise may have improved performance on the 10‐RM assessment, leading to increased workload. Conversely, increased familiarity with upper body resistance training likely also improves ACE performance. Thus, this method might be optimal for tailoring exercise intensity. Despite this, there was a limitation in the duration of the CARE test compared to the CCCT for participants with lower 10‐RM bicep curl weights (<15 lbs). For a few of these participants, the duration of the CARE test tended to be longer compared to the CCCT (Table 1). It is possible these smaller increases in workload may prolong the duration of exertion testing in athletes with concussion causing a blunted increase in the symptoms related to the test stage (Imhoff et al., 2017). Moreover, participants’ familiarity with cycling exercise over ACE may have influenced study results, potentially contributing to the individual variability observed since participants may have exhibited greater neuromuscular efficiency and more consistent physiological responses during the more familiar cycling protocol (CCCT) compared to the less familiar ACE protocol (CARE) (Waldron et al., 2016). Finally, the distribution of differences and association between means and differences in minute ventilation within stage violated assumptions for using a Bland–Altman analysis. Despite this, minute ventilation was retained in the analysis for consistency and since converging statistical analysis was performed assessing ICCs, providing a robust and convergent evaluation of agreement across multiple statistical techniques.

### 4.4 Conclusion

We found that the CARE test elicited lower HR, MCAv, $P_{\mathrm{ETC}O_{2}}$, ${\dot{V}}_{O_{2}}$ and ${\dot{V}}_{E}$ responses than the CCCT with individual variability generally increasing with exercise intensity. Despite this, the use of ACE during the CARE test elicited physiological exercise responses likely sufficient to exacerbate concussion symptoms and assess exercise tolerance during exertion testing. This investigation marks the beginning of a validation process to develop and implement a physiologically based upper‐body specific exercise protocol that will help inform the assessment and rehabilitation of Para athletes and individuals with lower‐limb impairments after concussion with future applications to more diverse populations Supplementary Material.

## AUTHOR CONTRIBUTIONS

Jonathan D. Smirl and Joshua J. Burkart conceptualized and designed the study. Jonathan D. Smirl., Joshua J. Burkart, Matthew G. Neill, Elizabeth K. S. Fletcher, Joel S. Burma, Nathan E. Johnson, performed the experiments. Jonathan D. Smirl., Joshua J. Burkart, Joel S. Burma, and Jean‐Michel Galarneau. conducted the data analysis. Jonathan D. Smirl. and Joshua J. Burkart wrote the manuscript. Matthew G. Neill, Elizabeth K. S. Fletcher, Joel S. Burma, Nathan E. Johnson, Jean‐Michel Galarneau, John J. Leddy, Mohammad N. Haider, William M. Adams, Cheri Blauwet, Chantel T. Debert, and Carolyn A. Emery. contributed to editing and revising the manuscript. All authors have approved the final version of the manuscript and agree to be accountable for all aspects of the work, ensuring that any questions related to the accuracy or integrity of any part of the work are appropriately investigated and resolved. All contributing and corresponding authors qualify for authorship, and all those who qualify for authorship are listed.

## CONFLICT OF INTEREST

None declared.

## Supporting information



Supporting Information

## Data Availability

Data from the article are available upon reasonable request to the corresponding author.

## 1

**TABLE A1:** Protocol for the Calgary Adapted aRm Ergometer (CARE) concussion exertion test showing watt/stage increases as a function of a participants 10 repetition maximum of bicep curl exercises.

10‐RM bicep curl (lbs)		
**Lower Limit**	**Upper Limit**	**Watt/Stage**
0	6.7	1
6.8	13.4	2
13.5	20.1	3
20.2	26.8	4
26.9	33.5	5
33.6	40.2	6
40.3	46.9	7
47.0	53.6	8
53.7	60.3	9
60.4	67.0	10

For every 6.7 lbs (3 kg) increase in a participants’ 10‐RM bicep curl weight, the CARE test protocol advises a wattage increase of 1 W. Each stage of the CARE test is 1 min in duration. lbs, pounds.
